# The use of teledentistry in clinical oral and maxillofacial pathology practice: an institutional experience

**DOI:** 10.3389/froh.2023.1063973

**Published:** 2023-07-20

**Authors:** Andres Flores-Hidalgo, John Collie, Shae King, Ford T. Grant, Nicole E. Beasley, Mark E. Moss, Thomas R. Tempel

**Affiliations:** ^1^ Division of Oral and Maxillofacial Pathology, East Carolina University School of Dental Medicine, Greenville, NC, United States; ^2^ Community Service Learning Center-Ahoskie, Department of General Dentistry, East Carolina University School of Dental Medicine, Greenville, NC, United States; ^3^ Department of Foundational Sciences, East Carolina University School of Dental Medicine, Greenville, NC, United States; ^4^ Department of Extramural Clinical Practices, East Carolina University School of Dental Medicine, Greenville, NC, United States

**Keywords:** oral lesions, teledentistry, telehealth, oral health, oral pathology, rural healthcare access

## Abstract

**Background:**

Although there has been a slight increase in dental professionals since 2011, 98 of North Carolina's 100 counties are designated as Dental Health Professional Shortage Areas by the Heath Resources and Service Administration. This shortage significantly increases disparities and access to primary and specialized oral health care. Also, dental professionals in these remote locations may feel the access and referrals to oral and maxillofacial pathologists cumbersome. In 2020, the COVID-19 pandemic prompted an inevitable surge in the use of digital technology due to the social distancing norms and lockdowns, which forced dental education institutions and practitioners to adjust to new ways of meeting, teaching, and providing dental care. In the present manuscript, we report our institutional experience delivering specialized dental care in rural areas.

**Materials and methods:**

We conducted a retrospective case series of diagnosis, management, and outcomes of patients who underwent synchronous or asynchronous virtual and remote examination of oral lesions at ECU School of Dental Medicine and one satellite clinic over seven years. For those cases that concluded on surgical sampling, the clinical impressions, differential diagnoses, and the final diagnosis were compared to assess the accuracy of the clinical exam through teledentistry.

**Results:**

The total study population consisted of 71 patients. Most of the remote consultations were done asynchronously. Also, most virtual clinical consultations were initiated due to clinical suspicion of malignancy and infectious/reactive conditions, accounting for 42% and 25.3% of all encounters.

**Conclusions:**

The presented data suggest how teledentistry can support clinical practice in rural areas to achieve optimal care for the patient in rural or remote communities. Also, it significantly decreases the travel required, the number of appointments, and increases the speed of diagnosis. Teledentistry is an excellent tool available to all clinicians and can dramatically aid in diagnosing oral mucosa lesions.

## Introduction

Teledentistry has been used for many years with different dental applications, and the benefits are indisputable. These include dental education of patients and students, access to specialized dental care in rural and remote areas, decreasing time between diagnoses and treatment, and reducing costs related to transportation (1). Previous studies have described the use of teledentistry cariology, oral pathology and oral medicine, endodontics, and oral radiology, as well as in dental education (2, 3).

It is well known that many dentists need to become more familiar with teledentistry and its many applications. Moreover, the general practitioner might find the detection process of oral lesions difficult, especially for potentially malignant mucosa disorders (4, 5). In this sense, consultations with specialists in oral pathology can render an accurate diagnosis, reduce the turnaround time between diagnosis and treatment, suggest surgical sampling of lesions for microscopic examination, and prompt patient referral for further treatment if necessary. This also directly impacts the early detection of oral cancer and increased access to specialized dental care in rural communities.

Teledentistry can support the oral health workforce. Despite several improvements in our dental care system, the implementation of new policies, and the increase in the number of dentists in the area, the state of North Carolina remains designated as a Dental Health Professional Shortage Area (DHPSA) by the North Carolina Health Professions Data System, where the state's dentist-per-population ratio is below 6 per 10,000 (6). According to the National Program of Cancer Registries, the observed incidence of oral and oropharyngeal cancer in North Carolina has increased from an observed rate of 10.7 in 2002 to 12.8 per 10,000 residents. Moreover, the North Carolina Center Cancer registry estimates approximately 1,570 new oral and oropharynx cancer cases in 2022 (7). Hence, it is still difficult to access dental services for many patients in our state, making early diagnosis and treatment of oral diseases and early detection of oral cancer even more challenging.

The ECU School of Dental Medicine (ECU SoDM) is very well connected via its unique educational and patient care model that includes eight Community Service-Learning Centers (CSLCs) or satellite clinics across the state in rural areas each of which has been utilizing telehealth for remote virtual lectures, seminars, and provider-to-provider virtual consultations for endodontics and oral and maxillofacial pathology. The application of this technology can extend general and specialized medical and dental care to patients who reside in rural or remote areas and support the local clinician in providing optimal care in diagnosing and managing oral pathoses. This manuscript aims to report our institutional experience using synchronous and asynchronous teledentistry at one of the CSLC locations (ECU SoDM Ahoskie CSLC) for assessing oral lesions and to assess the accuracy of identification and management of oral lesions through teledentistry.

## Materials and methods

This study was conducted as a retrospective case series, of diagnosis, management and outcomes of patients who underwent virtual and remote examination of oral lesions at ECU SoDM Ahoskie CSLC and ECU SoDM Ross Hall (main campus). The study was registered and approved by the ECU Institutional Review Board under the number UMCIRB 22-000936. All study procedures were performed in accordance with the Helsinki and Research Committee Regulations.

**Figure 1:**
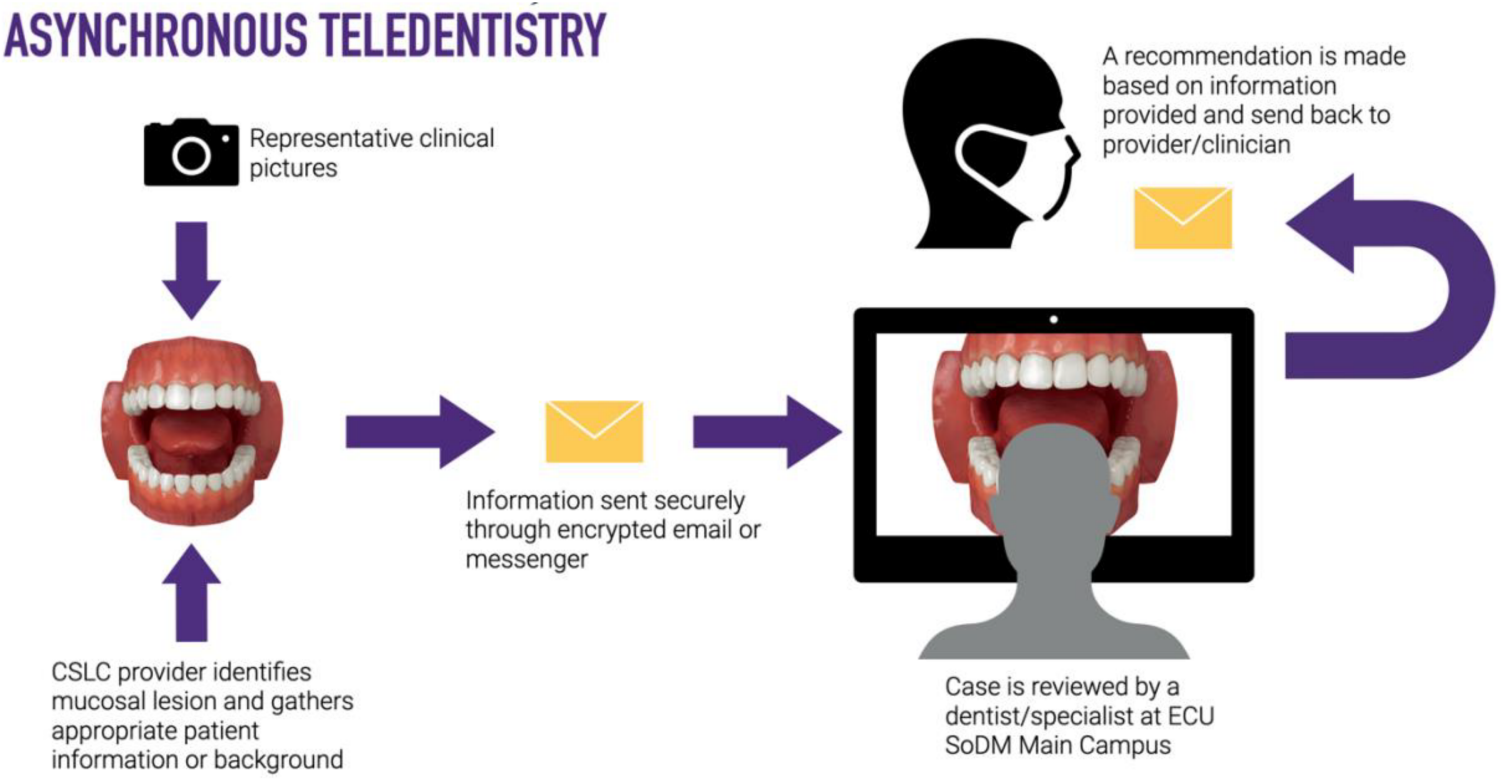
Diagram of an asynchronous consultation.

**Figure 2:**
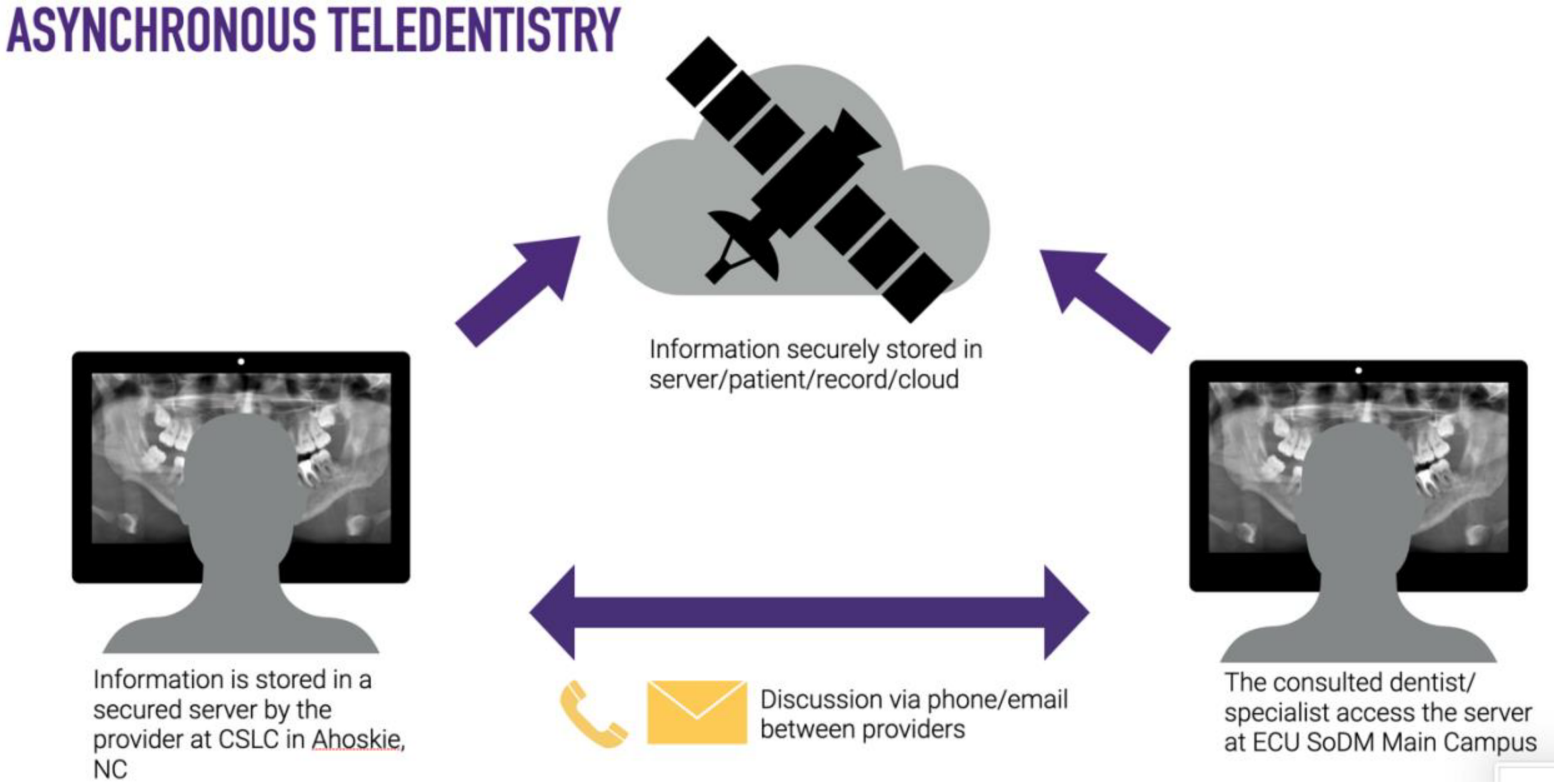
Diagram of the “store-and-forward” workflow for asynchronous and synchronous consultations.

**Figure 3:**
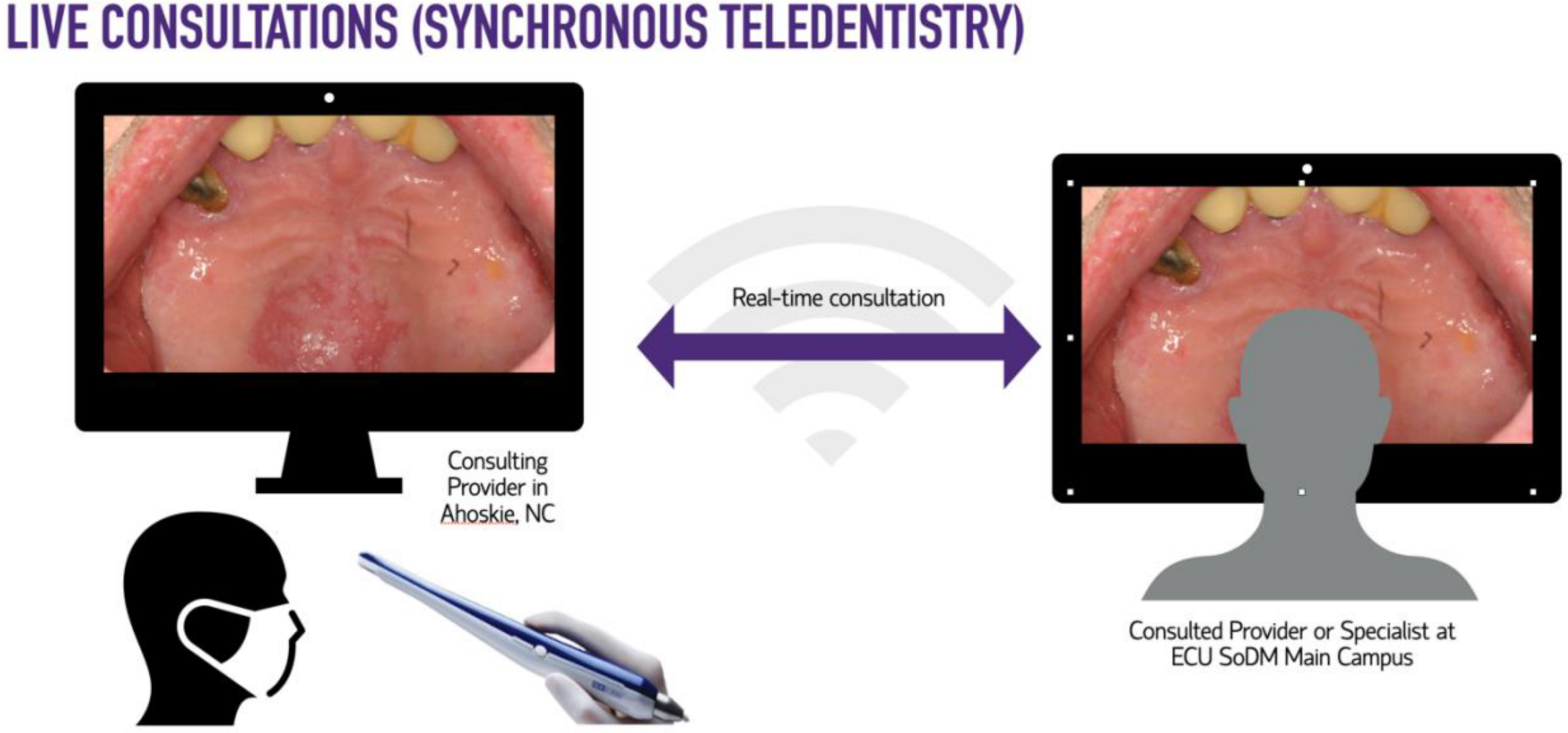
Workflow diagram of synchronous or “real-time” consultations.

**Figure 4:**
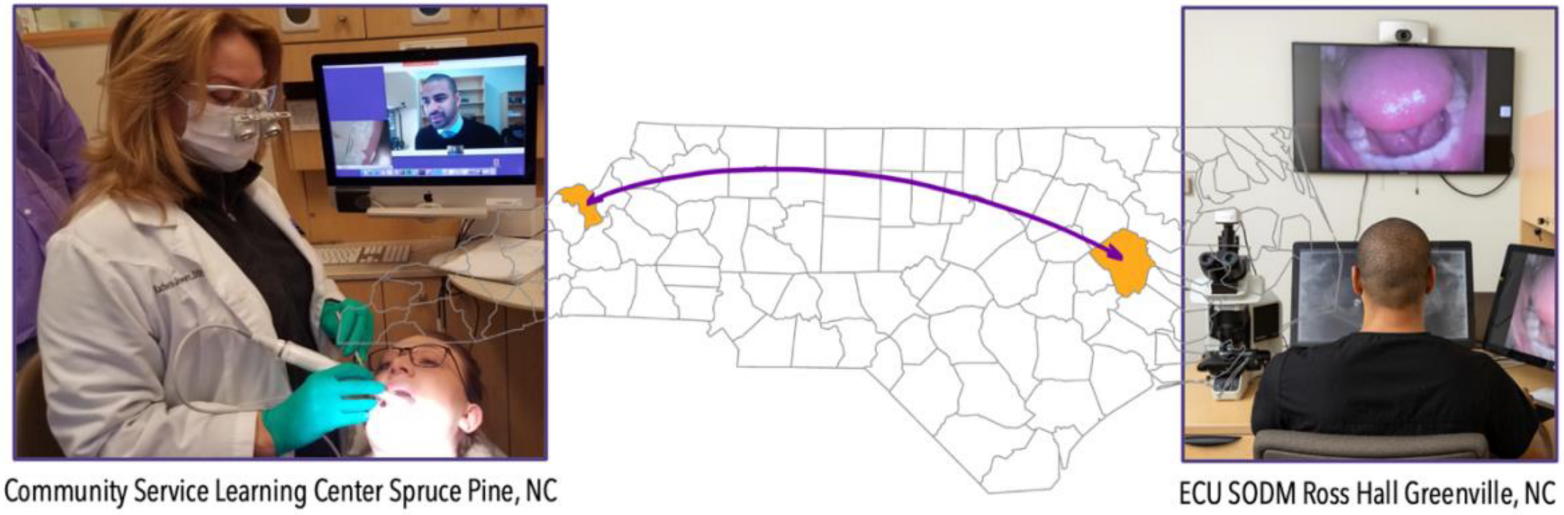
Example of synchronous “provider-to-provider “consultation between a satellite clinic in Spruce Pine, NC and Main Campus in Greenville, NC (313 miles apart).

For this study, synchronous teledentistry is considered a “real-time” consultation or communication between two dental providers while the patient is in the dental chair. The consultees included General Dentistry Faculty, Advanced Education in General Dentistry Residents, and Dental Students. The consulted providers included Oral Surgery, Oral Pathology, and Oral Radiology specialists. The consulting clinician uses an intraoral device (DEXcam™ 4 HD. DEXIS™, PA, USA or SOPRO 617. Norwich, UK), connected endodontic microscope (Global G3 Surgical Microscope. Global™, MO, USA) or extraoral device such as InVision Teleconsultation camera (Enovate Medical LLC. Tennessee, USA) for the remote transmission of imaging in real-time. Asynchronous teledentistry is remote communication where a clinician is approached via secure email or an electronic patient record messaging system after the patient's appointment. Based on the information provided by the clinician (clinical pictures, radiographs, a case summary, clinical impression, medical, surgical, and social history), the consultant will respond with the lesion's presumed nature, a working diagnosis, and recommended management. The turnaround time per consultation was usually 24–48 h. Figures 1–4 exemplify our clinics’ workflow of asynchronous and synchronous talks.

All patients with an oral mucosal or radiographic lesion of concern were remotely seen (by synchronous or asynchronous teledentistry) over seven years between January 1, 2014 to December 31, 2021, and were included in the study. The evaluation of the management of oral lesions was conducted using Donabedian's framework process improvement system, where we focused on the modality of care delivered, the type of care, and effect of teledentistry in the dental care delivered to our patients. The paper clinic biopsy log was cross-referenced with all biopsy procedural and billing codes and surgical pathology documentation. For those cases where surgical sampling of the lesion(s) was performed, the clinical impressions or differential diagnoses, and the final microscopic diagnosis were compared to assess the accuracy of the clinical exam through teledentistry, utilizing SPSS software for F-score statistical analysis to evaluate whether the variances of the two groups are equal or not (IBM Corp. Released 2021. IBM SPSS Statistics for Windows, Version 28.0. Armonk, NY: IBM Corp) (8). All biopsies were interpreted by a board-certified oral and maxillofacial pathologist.

Other variables collected in the study included the modality of teledentistry utilized (synchronous vs. asynchronous), patient's home county (urban vs. rural), type of consulting provider (dental student, AEGD resident, or faculty), clinical description and initial differential diagnoses, microscopic diagnosis, the time between consult and treatment, and outcome, if available. The time between consultation and treatment rendered was measured as the days between the remote or virtual consultation and the day of the biopsy, if applicable.

## Results

The total study population consisted of 71 patients. The age of the patients ranged between 10 and 82 years of age (mean 57), and 63% of the study population were males, making the male-to-female ratio of consulted patients 2:1. Most of the remote consultations were done asynchronously (store-and-forward) using clinical pictures or radiographs. All patients were determined to come from or live in zip codes denominated in rural areas. 80% of all consultations were performed by dental residents, faculty members, and dental students.

For analysis, the clinical impressions, or reason for consultation with oral pathology, were classified as suspicious for malignancy or potentially malignant, immune-mediated disorders, developmental lesions, viral and infectious or reactive. Potentially malignant and infections/reactive conditions were predominant, 42% and 25.3%, respectively. This category was followed by developmental (odontogenic and non-odontogenic) lesions, with accounted for 21.1% of all encounters; this category includes odontogenic cysts and tumors not associated with odontogenic infections. The clinical appearance of oral lesions in each category and the number of encounters can be found on display in Table 1.

**Table 1:** Number and percentages of differential diagnoses or reason for the teledentistry consultation with remote oral and maxillofacial pathology.

Clinical concerns/differential diagnoses	Cases *n* [%]
Rule out malignancy or suspicious for malignancy, e.g.,: • “Rule out malignancy” • “leukoplakia” • “erythroleukoplakia”	24 [42%]
Immune-mediated conditions, e.g.,: • “Lichenoid reaction vs. lichen planus” • “Rule out lichenoid reaction” • “Desquamative gingivitis”	2 [3.5%]
Developmental odontogenic or not odontogenic, e.g.,: • “OKC vs. ameloblastoma” • “Radiolucent lesion not associated with surrounding dentition” • “Painless swelling on the hard palate”	12 [21.1%]
Viral, e.g.,: • “Cauliflower-like lesion” • “Papillomatous lesion” • “Papillary mass in the posterior palate”	4 [7%]
Infectious/reactive, e.g.,: • “Upper lip swelling following trauma” • “Nodule on lower mucosal lip” • “Periapical radiolucency”	15 [26.3%]

Of all reported cases, 57 involved surgical sampling of the specimen (incisional or excisional biopsy with microscopic examination). For these 57 cases, after reviewing the clinical findings, taking a detailed medical history, and, if necessary, performing additional diagnostic tests, the decision to perform a diagnostic surgical biopsy is made between both provider (in-house and remote) and the patient. The mean time between the initial consultation and incisional or excisional biopsy was 9.6 days. The final diagnoses or biopsy results were classified into seven categories: malignant neoplasms, benign neoplasms, premalignant mucosa conditions, infectious lesions, reactive, developmental, and immune-mediated diseases. Over thirty-six percent (36.84%) of all diagnoses rendered were reactive, encompassing pyogenic granulomas, traumatic ulcerative granuloma with stromal eosinophilia (chronic ulcers), and traumatic fibromas, among others. Malignant neoplastic lesions accounted for 8 cases of the data reviewed. A summary of biopsy results arranged in categories can be found in Table 2. Accuracy measurements between the clinical impression and final microscopic diagnosis were performed with an *F*-score of 0.80.

**Table 2:** Categorized final diagnosis for cases in which a teledentistry consultation ended in surgical sampling of the lesion(s). Below each category are examples of the diagnoses rendered and included in each category.

Final diagnoses after microscopic examination	Cases *n* [%]
Neoplastic (malignant)/premalignant • Oral epithelial dysplasia • Adenoid cystic carcinoma	8 [14%]
Infectious • Radicular cyst • Periapical granuloma • Actinomycosis • Candidiasis	12 [21%]
Reactive • Mucocele • Traumatic ulcerative granuloma with stromal eosinophilia • Pyogenic granuloma • Gingival fibroma	21 [36.8%]
Developmental (odontogenic or non-odontogenic) • Dentigerous cyst • Lateral periodontal cyst • Nasopalatine duct cyst	6 [10.5%]
Neoplastic (benign) • Hemangioma • Odontoma	6 [10.5%]
Immune-mediated • Lichen planus • Lichenoid mucositis	4 [7%]

## Discussion

Teledentistry services have enhanced the value of ECU SoDM, as they have established professional relationships among providers, maintained a bridge of communication between SoDM and the faculty members at our CSLCs, improved the quality and availability of oral care services for rural patients, and decreased diagnosis time in the patient requiring diagnostic services from the Division of Oral and Maxillofacial Pathology. Our program demonstrates that teledentistry consultations assist access to specialized dental care; it does not require complex procedures or sophisticated infrastructure.

Similar studies have reported their clinical experience using virtual images and radiographs for virtual and remote consultations in diagnosing oral lesions (5, 9, 10). It appears that telediagnoses in clinical oral and maxillofacial pathology can be a powerful tool for patients, although face-to-face evaluations are the best for establishing a definitive diagnosis. The literature also reports that high-resolution clinical pictures and a detailed summary of the case can help prioritize referral requests, particularly in the case of high suspicion for a malignant process (11–14).

This is particularly important, as most lesions of our sample in this category were encountered in older adults. Teledentistry is of greater importance in the aging adult population and for patients with reported health disparities or lack of access to dental care. For example, the 2018 North Carolina Health Equity Report by the North Carolina Department of Health and Human Services states that in 2016, 32% of white adults in North Carolina did not visit a dentist or dental clinic. Additionally, 44% of African Americans and 51% of Hispanics/Latinx did not see a dentist at the same timeframe. The lack of dental professionals in rural areas travel required to urban centers, and the cost of care are the main drivers behind this data (15, 16). Teledentistry can help alleviate access to care issues due to decreased mobility, lack of transportation, and physical and social isolation in rural areas (17).

The biopsy results were predominantly benign lesions with etiology mainly associated with irritative and infectious factors such as pyogenic granulomas, traumatic fibromas, and hyperkeratosis, among others. However, most virtual clinical consultations were initiated due to clinical suspicion of malignancy. These results agree with other studies reported in the literature where the leading utility for teledentistry has been reported as a powerful screening tool for oral mucosal lesions and early detection of oral cancer, which is supported by the accuracy score reported (10, 13, 18).

**Figure 5:**
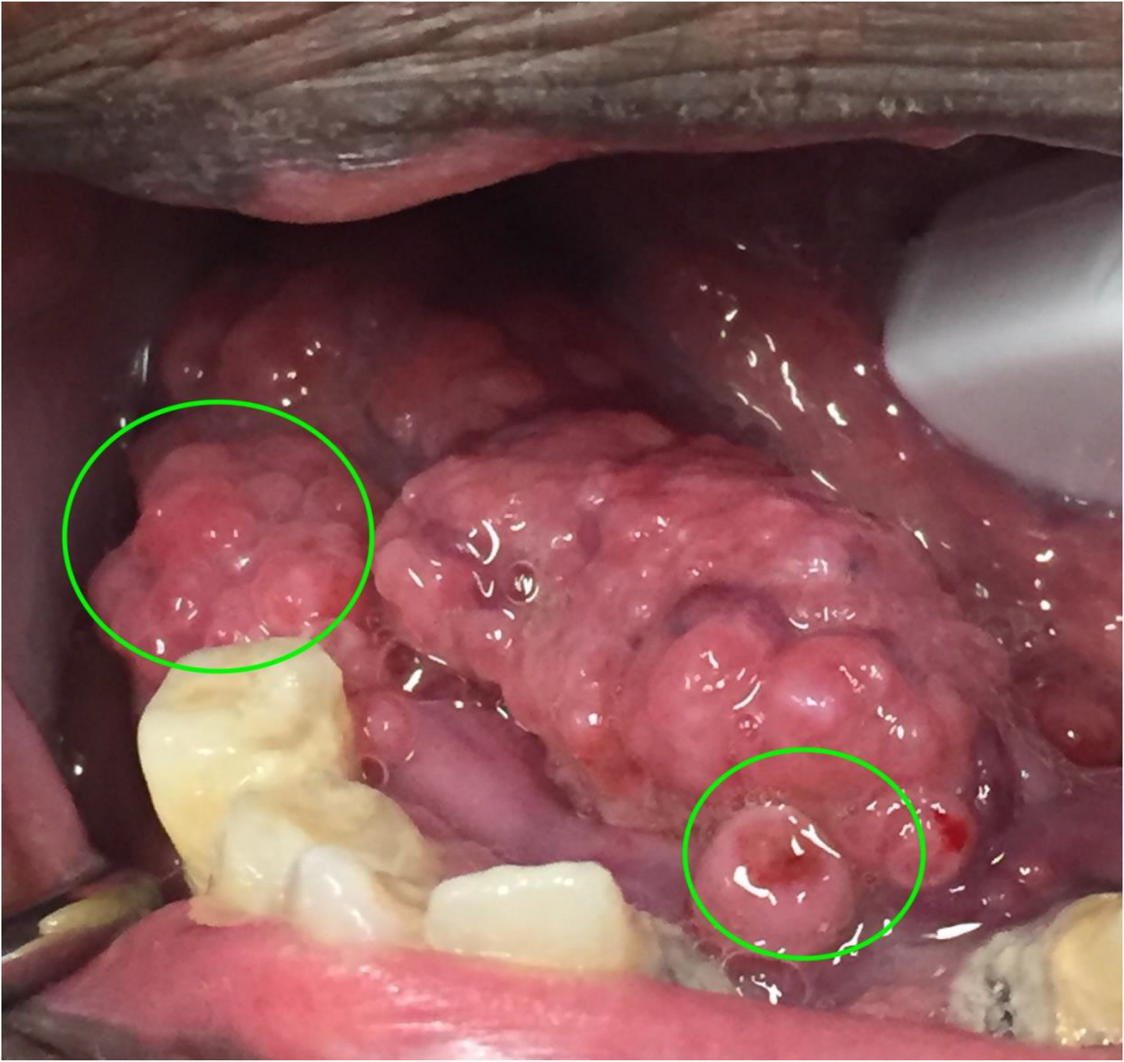
Biopsy sites suggested remotely by the oral and maxillofacial pathologist during a synchronous teledentistry consultation. Image taken from: Bankhead et al. (20).

A total of 8 (14%) cases corresponded to neoplastic malignancy or potentially malignant lesions reinforcing the results presented by Petruzzi and De Benedittis (19), which involved a middle-aged African American patient with a mass on the floor of their mouth (Figure 5). It is also important to highlight that the incisional biopsy performed on this patient and the initial management of the patient was conducted by a senior dental student under the supervision of a faculty member on-site and a remote oral pathologist who was consulted via teledentistry (20). This also highlights the educational and research component involved and the increased exposure to specialized dental care that teledentistry offers our students. Similar experience and the potential for remote education was highlighted and explored initially by the US Armed Forces and even more recently by other groups after the COVID-19 pandemic (21–23).

In our experience, using intraoral and high-resolution extraoral cameras can also significantly increase access to care for patients in rural communities for specialized care. Teledentistry or telediagnosis is not intended to replace conventional consultations but rather offer support in clinical oral pathology when the general practitioner is unsure and helps diagnose and manage patients. This support is critical in rural locations where patients must travel long distances to consult an oral and maxillofacial pathologist or oral medicine specialist. All the patients involved in this study were from a rural location in Eastern North Carolina, in or surrounding Hertford County. This is important because fewer than five dental professionals in rural counties provide services for a population of over 4,000 habitats. To the best of our knowledge, there are no practicing oral and maxillofacial pathologists or oral medicine specialists in the specified area, which means that our CSLC is the only dental service in the area that provides access to this type of specialized dental care in the area.

Limitations of our study, such as its retrospective nature, underreporting of remote consultation that did not result in surgical sampling, and the involvement of multiple providers in the data collection over the years, need to be considered. The fact that our study does not comprise a control group is also a limitation. Still, the suitability of performing a controlled study on our patient population, and institution, is limited.

## Conclusion

Increasing efficiency in the early detection of oral lesions by dentists using teledentistry is an essential step toward early diagnosis. In this sense, teledentistry allows specialists distant from patients to diagnose and treat oral lesions, removing geographical barriers and providing an opportunity for early diagnosis, which is essential for malignant and potentially malignant lesions. Future studies should investigate the economic impact of offering this service to our patients. Also, further studies comparing this data to face-to-face clinical examinations and surveys to assess dental provider satisfaction are warranted.

## Data Availability

The raw data supporting the conclusions of this article will be made available by the authors, without undue reservation.
